# Hepatitis B vaccine birth dose coverage among hepatitis B-exposed and hepatitis B-unexposed infants: evidence from the Healthy Beginning Initiative program in Benue State, Nigeria

**DOI:** 10.11604/pamj.2024.47.67.40466

**Published:** 2024-02-14

**Authors:** Babayemi Oluwaseun Olakunde, Ijeoma Uchenna Itanyi, Tonia Chinyelu Onyeka, Elijah Paintsil, Kwasi Torpey, Nwamaka Lasebikan, Chibuike Ogwuegbu Chigbu, Echezona Edozie Ezeanolue

**Affiliations:** 1 Department of Community Prevention and Care Services, National Agency for the Control of AIDS, Abuja, Nigeria,; 2 Center for Translation and Implementation Research, University of Nigeria, Nsukka, Enugu, Nigeria,; 3 Department of Community Medicine, College of Medicine, University of Nigeria, Nsukka, Enugu, Nigeria,; 4 Department of Anesthesia/Pain and Palliative Care Unit, College of Medicine, University of Nigeria, Nsukka, Nigeria,; 5 Department of Pediatrics, Yale School of Medicine, New Haven, CT 06510, USA,; 6 Department of Epidemiology of Microbial Diseases, Yale School of Public Health, New Haven, CT 06510, USA,; 7 Department of Pharmacology, Yale School of Medicine, New Haven, CT, USA,; 8 Department of Population, Family and Reproductive Health, School of Public Health, University of Ghana, Legon-Accra, Ghana,; 9 Department of Radiation Medicine, College of Medicine, University of Nigeria, Nsukka, Enugu, Nigeria,; 10 Department of Obstetrics and Gynaecology, College of Medicine, University of Nigeria, Nsukka, Enugu, Nigeria,; 11 Department of Paediatrics, College of Medicine, University of Nigeria, Nsukka, Enugu, Nigeria,; 12 Healthy Sunrise Foundation, Nevada, USA

**Keywords:** Infants, immunization, perinatal transmission, viral hepatitis

## Abstract

**Introduction:**

Nigeria offers universal hepatitis B birth-dose vaccine (HepB-BD) for the prevention and control of hepatitis B (HepB). While prior studies suggest low coverage of HepB-BD in Nigeria, there is a paucity of evidence on the association between the uptake of HepB-BD and maternal HepB status. This study aimed to determine HepB-BD coverage and the associated factors among infants of HepB-positive and -negative women in Nigeria.

**Methods:**

the study was a secondary analysis of data from the Healthy Beginning Initiative program conducted between June 2016 and October 2018 in Benue State, Nigeria. The analysis was restricted to data from a cohort of 6269 mothers who had HepB screening during pregnancy and completed the HepB infant immunization question in the post-delivery survey. The association between the coverage of HepB-BD and maternal HepB status, sociodemographic characteristics, and obstetric factors were determined using crude and adjusted relative risks.

**Results:**

about 10% of the women tested HepB positive. The coverage of HepB-BD was 64% (63.2% among infants of HepB-positive mothers and 63.8% among HepB-negative mothers). The likelihood of infants of HepB-positive mothers receiving HepB-BD was not significantly different from infants of HepB-negative mothers (aRR=0.97, 95%CI= 0.92-1.04). Among HepB-positive mothers, infants of mothers younger than 20 years (aRR=1.49, 95%CI=1.03-2.16) or those who received antenatal care (aRR=1.41, 95%CI=1.16-1.71) were more likely to receive HepB-BD, while mothers with no previous pregnancies (aRR=0.73, 95%CI=0.59-0.91) were less likely to receive HepB-BD. Among HepB-negative mothers, infants of less-educated mothers were less likely to receive HepB-BD (aRR=0.96, 95%CI=0.92-0.99), whereas infants of mothers who received antenatal care (aRR=1.23, 95%CI=1.16-1.31) or had an institutional delivery were more likely (aRR=1.29, 95%CI=1.23-1.36) to receive HepB-BD. **Conclusion:** our findings highlight the need to improve HepB-BD uptake, particularly among HepB-exposed infants who are at risk of perinatal transmission of HepB.

## Introduction

About 2 billion people are estimated to have been infected with hepatitis B (HepB) infection and over 250 million people are living with the chronic infection, globally [1,2]. The burden of HepB varies geographically, with high endemicity in many African countries [3,4]. Nigeria has the largest burden of HepB in Africa, with over 20 million estimated to be infected with HepB [4]. As in other African countries, the majority of these infections are acquired in infancy and early childhood through perinatal or horizontal transmission [5]. Without intervention, 90% of infants born to HepB-positive mothers who have high viral load and/or are positive for the hepatitis B e antigen (HBeAg) are at risk of being infected [6], and about 80-90% of infants infected with HepB through perinatal transmission develop chronic liver disease [6,7].

Although HepB is vaccine-preventable, the low uptake of vaccination has hampered its prevention and control in Nigeria. As recommended by the World Health Organization (WHO) [8], Nigeria´s national programme on immunization includes a monovalent hepatitis B vaccine birth-dose (HepB-BD) administered within 24 hours of birth, followed by three doses of hepatitis B vaccine (as part of the pentavalent vaccine) at 6, 10, and 14 weeks to prevent the acquisition of HepB infection [9]. HepB-BD reduces the risk of vertical transmission to less than 1% among infants of HBeAg-negative mothers and 20-30% among infants of HBeAg-positive mothers [10]. However, the coverage of HepB-BD has been suboptimal in Nigeria; less than the 80% target coverage by the National Strategic Plan for Viral Hepatitis Control in Nigeria (2016-2020) [11]. In 2019, only 52% of babies were estimated to have received HepB-BD [12]. The reasons for the low coverage of HepB-BD have been understudied in Nigeria, but available studies have indicated that poor knowledge of HepB vaccine, unavailability of HepB vaccines, high cost of vaccination, long distance to health facilities, lack of antenatal care, and high rates of home deliveries affect the uptake and timeliness of HepB-BD in Nigeria [13-15].

Despite the growing literature on HepB-BD in Nigeria, there is a lack of evidence on how the uptake is influenced by maternal HepB status. While Nigeria offers free, universal HepB-BD for newborns, higher coverage among HepB-exposed babies is desirable, considering the risk of transmission. The objective of this study was to determine HepB-BD coverage and the associated factors among infants of HepB-positive and -negative women in Benue State, Nigeria.

## Methods

**Study design, setting, and participants:** this study was a secondary analysis of data from the Healthy Beginning Initiative (HBI) program conducted between June 2016 and October 2018 in 80 churches across 12 of the 23 local government areas (LGAs) in Benue State, Nigeria. Benue State in located in the North Central region of Nigeria with a projected population of 5,741,8155 in 2016 based on the 2006 population estimates. At 67.7% coverage, Benue State has the third highest HepB-BD coverage after Nasarawa State (84.7%) and Federal Capital Territory (83.0%) and in the North Central zone [12]. The HBI is a culturally adapted, congregational-based program designed to identify pregnant women, implement health interventions, and support linkage to health services for the cohort of pregnant women and their families. Details of the HBI program have been described elsewhere [16,17]. In brief, it has three main components: (i) prayer sessions (the priest or pastor prays for pregnant women and their male partners for a healthy pregnancy and safe delivery, and encourages pregnant women to seek antenatal care at a health facility); (ii) baby shower (a reception and health fair is held in churches for the provision of health education on antenatal care and onsite integrated testing for HIV, hepatitis B, and sickle cell genotype for pregnant women and their male partners); and (iii) baby reception (organized at six to eight weeks post-delivery for women who participate in the baby shower, to provide health education on postnatal care and also for the completion of a post-delivery questionnaire). Over 10,000 pregnant women participated in the HBI.

**Data collection:** during the baby showers, sociodemographic data, such as age, sex, marital status, education, occupation, monthly income, and distance to a health facility were obtained from the participants using the enrolment questionnaire. In addition to other screening tests, HepB rapid tests were also performed onsite with Global rapid HBsAg antibody test strips. The rapid test kit has a sensitivity of >99.9% and a specificity of 99.4%, and it has been approved for use in Nigeria by the National Agency for Food and Drug Administration and Control (NAFDAC Reg No: 03-1192) [18]. Participants who tested positive for HBsAg were referred to a health facility for care. As part of the post-delivery survey at the baby receptions, the women were asked if their babies received the HepB vaccine at birth.

**Variables:** the outcome variable was the receipt of HepB-BD (yes/no). The independent variables included the mother´s HepB status (HepB positive/HepB negative) and the following sociodemographic and obstetric factors that may likely influence the uptake of childhood vaccination: age, educational attainment, income, distance to a health facility, previous pregnancies, antenatal care, and place of delivery [19-23].

**Data analysis:** the analysis was restricted to 6269 mothers who had HepB screening during pregnancy (baby shower) and completed the HepB infant immunization question after delivery (baby reception). The quantitative data was summarized using descriptive statistics and the association between the receipt of HepB-BD and the independent variables were determined using crude and adjusted relative risks. The relative risks were estimated by fitting univariate and multivariable log-binomial regression models. The adjusted model included all the independent variables regardless of their significance level in the univariate analysis. The sociodemographic and obstetric factors associated with the receipt of HepB-BD stratified by the mother´s HepB status were also examined using log-binomial regression models. We performed a complete case analysis. A p-value of <0.05 was considered statistically significant. The data analysis was performed with IBM® SPSS® Statistics 26.

**Ethical consideration:** the HBI was approved by the Health Research Ethics Committee of the University of Nigeria Teaching Hospital, Enugu, Nigeria. Although consent was not needed to participate in the baby showers, written informed consent was obtained from the participants for laboratory tests and questionnaire data collection.

## Results

Table 1 shows the characteristics of the women included in the study. Approximately 10% of them were HepB-positive. The majority of them (81%) were between 20 and 34 years and about 54% had at least secondary education. Over 90% earned a monthly income of less than ₦20,001 ($55), while 74% resided less than 6km away from a health facility. Thirty percent of the women had had five or more pregnancies before the index pregnancy. About 74% received antenatal care and 65% delivered in a health facility.

**Table 1:** characteristics of the study population, Healthy Beginning Initiative program, Benue State, Nigeria (N=6269)

Mother's characteristics	N (%)
**HepB status**	
Negative	5628 (89.8)
Positive	641 (10.2)
**Age in years**	
<20	842 (13.4)
20-34	5067 (80.8)
≥35	360 (5.7)
**Educational attainment**	
None^a^ or primary	2899 (46.3)
Secondary or higher	3367 (53.7)
Missing	3
**Monthly income^b^**	
₦0-₦20,000	5786 (92.3)
₦20,001-₦50,000	378 (6.0)
≥₦50,001	104 (1.7)
Missing	1
**Distance to a health facility**	
0-5 kilometers	4638 (74.0)
6-10 kilometers	1417 (22.6)
≥11 kilometers	214 (3.4)
**Previous pregnancies^c^**	
0	992 (15.8)
1-3	3408 (54.4)
≥4	1869 (29.8)
**Antenatal care**	
No	1662 (26.5)
Yes	4604 (73.5)
Missing	3
**Place of delivery**	
Non-institutional	2216 (35.4)
Institutional	4038 (64.6)
Missing	15

a No formal education; ^b^₦361~$1(USD); ^c^number of previous pregnancies before the current pregnancy at the time of this study; HepB= hepatitis B

Overall, 64% of the women reported that their infants received HepB-BD (Table 2). Although the receipt of HepB-BD varied by the respondents´ characteristics, it was similar among the infants of HepB-positive mothers (63.2%) and HepB-negative mothers (63.8%) (Table 2). Compared with HepB-negative mothers, there was no statistically significant difference in the likelihood of infants of HepB-positive mothers receiving HepB-BD compared to HepB-negative mothers (Crude Relative risk [RR]=0.99, 95%CI= 0.93-1.06) (Table 3). The adjusted model also showed no significant difference between the two groups of women (adjusted relative risk [aRR]=0.97, 95%CI=0.92-1.03), after controlling for age, educational attainment, income, distance to a health facility, previous pregnancies, antenatal care, and the place of delivery (Table 3). In the adjusted model, infants of mothers with primary or no formal education were less likely to receive HepB-BD compared with mothers who had secondary or higher education (aRR=0.95, 95%CI=0.91-0.99) (Table 3). However, infants of mothers who received antenatal care (aRR=1.25, 95%CI=1.18-1.32) or who had an institutional delivery (aRR=1.29, 95%CI=1.23-1.36) were more likely to receive HepB-BD, compared with infants whose mothers that did not receive antenatal care or have an institutional delivery, respectively (Table 3).

**Table 2:** receipt of hepatitis B birth-dose vaccine by mother’s characteristics, Healthy Beginning Initiative program, Benue State, Nigeria

Mother's characteristics	Did not receive HepB-BD	Received HepB-BD	P-value^b^
	N (%)	N (%)	
**HepB status**			0.064
Negative	2038 (36.2)	3590 (63.8)	
Positive	235 (36.7)	490 (63.3)	
**Age in years**			
<20	335 (39.8)	507 (60.2)	
20-34	1805 (35.6)	3262 (64.4)	
≥35	133 (36.9)	227 (63.1)	
**Educational attainment**			<0.001
None or primary	1173 (40.5)	1726 (59.5)	
Secondary or higher	1099 (32.6)	2268 (67.4)	
**Monthly income^a^**			0.021
₦0-₦20,000	2125 (36.7)	3661 (63.3)	
₦20,001-₦50,000	113 (29.9)	265 (70.1)	
≥₦50,001	34 (32.7)	70 (67.3)	
**Distance to a health facility**			0.965
0-5 kilometers	1685 (36.3)	2953 (63.7)	
6-10 kilometers	512 (36.1)	905 (63.9)	
≥11 kilometers	76 (35.5)	138 (64.5)	
**Previous pregnancies**			0.236
0	356 (35.9)	636 (64.1)	
1-3	1210 (35.5)	2198 (64.5)	
≥4	707 (37.8)	1162 (62.2)	
**Antenatal care**			<0.001
No	852 (51.3)	810 (48.7)	
Yes	1419 (30.8)	3185 (69.2)	
**Place of delivery**			<0.001
Non-institutional	1094 (49.4)	1122 (50.6)	
Institutional	1171 (29.0)	2867 (71.0)	
All	2273 (36.3)	3996 (63.7)	

a ₦361~$1(US dollars); ^b^Chi-square test; HepB= hepatitis B; HepB-BD= hepatitis B birth-dose vaccine

**Table 3:** factors associated with the receipt of hepatitis B birth-dose vaccine, Healthy Beginning Initiative program, Benue State, Nigeria

Mother's characteristics	Univariate	Multivariable
	RR (95% CI)	aRR (95%CI)
**HepB status**		
Negative	1	1
Positive	0.99 (0.93-1.06)	0.97 (0.92-1.03)
**Age in years**		
≥35	1	1
20-34	1.02 (0.94-1.11)	0.99 (0.91-1.08)
<20	0.96 (0.87-1.05)	0.95 (0.86-1.05)
**Educational attainment**		
Secondary or higher	1	1
None or primary	0.88 (0.85-0.92)***	0.95 (0.91-0.99)**
**Monthly income^a^**		
≥₦50,001	1	1
₦20,001-₦50,000	1.04 (0.90-1.21)	1.04 (0.91-1.20)
₦0-₦20,000	0.94 (0.82-1.08)	0.98 (0.86-1.11)
**Distance to a health facility**		
≥11 kilometers	1	1
6-10 kilometers	0.99 (0.89-1.10)	1.00 (0.91-1.11)
0-5 kilometers	0.99 (0.89-1.09)	1.00 (0.91-1.10)
**Previous pregnancies**		
≥4	1	1
1-3	1.04 (0.99-1.08)	1.01 (0.97-1.06)
0	1.03 (0.97-1.09)	1.00 (0.94-1.06)
**Antenatal care**		
No	1	1
Yes	1.42 (1.35-1.50)***	1.25 (1.18-1.32)***
**Place of delivery**		
Non-institutional	1	1
Institutional	1.40 (1.34-1.47)***	1.27 (1.21-1.34)***

a ₦361~$1(US dollars); ** p <0.01; *** p<0.001; HepB= hepatitis B; RR= risk ratio; aRR= adjusted risk ratio; CI= confidence interval

Table 4 shows the associations between the mother´s sociodemographic and obstetric characteristics and the receipt of HepB-BD stratified by HepB status. Among HepB-positive mothers, age, previous pregnancies, and receiving antenatal care were associated with the uptake of HepB-BD. Infants of HepB-positive mothers less than 20 years ( versus ≥35 years) (aRR=1.49, 95%CI=1.03-2.16) or HepB-positive mothers who received antenatal care (versus no antenatal care) (aRR=1.41, 95%CI=1.16-1.71) were more likely to receive HepB-BD, while HepB-positive mothers with no previous pregnancies (versus ≥4 previous pregnancies) were 27% less likely to receive HepB-BD (aRR=0.73, 95%CI=0.59-0.91)

**Table 4:** factors associated with the receipt of hepatitis B birth-dose vaccine by mother’s hepatitis B status, Healthy Beginning Initiative program, Benue State, Nigeria

Mother’s characteristics	HepB positive		HepB negative	
	Univariate	Multivariate	Univariate	Multivariate
	RR (95%CI)	aRR (95%CI)	RR (95%CI)	aRR (95%CI)
**Age in years**				
≥35	1	1	1	1
20-34	1.28 (0.92-1.78)	1.28 (0.92-1.80)	1.00 (0.92-1.09)	0.97 (0.89-1.05)
<20	1.32 (0.92-1.89)	1.49 (1.03-2.16)*	0.92 (0.84-1.02)	0.91 (0.82-1.01)
**Educational attainment**				
Secondary or higher	1	1	1	1
None or primary	0.87 (0.76-0.97)*	0.89 (0.80-1.00)	0.89 (0.85-0.92)***	0.96 (0.92-0.99)*
**Monthly income^a^**				
≥₦50,001	1	1	1	1
₦20,001-₦50,000	1.14 (0.63-2.05)	1.03 (0.58-1.83)	1.03 (0.89-1.20)	1.05 (0.91-1.21)
₦0-₦20,000	1.17 (0.68-2.01)	1.12 (0.66-1.92)	0.92 (0.80-1.06)	0.96 (0.85-1.10)
**Distance to a health facility**				
≥11 kilometers	1	1	1	1
6-10 kilometers	1.11 (0.74-1.67)	1.18 (0.79-1.74)	0.98 (0.88-1.09)	0.98 (0.89-1.09)
0-5 kilometers	1.18 (0.80-1.74)	1.24 (0.85-1.81)	0.97 (0.87-1.08)	0.97 (0.88-1.07)
**Previous pregnancies**				
≥4	1	1	1	1
1-3	1.01 (0.89-1.15)	0.93 (0.82-1.05)	1.04 (0.99-1.09)	1.02 (0.97-1.07)
0	0.82 (0.65-1.02)	0.73 (0.59-0.91)**	1.06 (0.99-1.12)	1.03 (0.97-1.10)
**Antenatal care**				
No	1	1	1	1
Yes	1.48 (1.24-1.77)***	1.41 (1.16-1.71)**	1.41 (1.34-1.49)***	1.23 (1.16-1.31)***
**Place of delivery**				
Non-institutional	1	1	1	1
Institutional	1.24 (1.08-1.43)**	1.06 (0.91-1.22)	1.42 (1.36-1.49)***	1.29 (1.23-1.36)***

a ₦361~$1(US dollars); * p <0.05; ** p <0.01; ** p<0.001; HepB= hepatitis B; RR= risk ratio; aRR= adjusted risk ratio; CI= confidence interval

However, among HepB-negative mothers, educational attainment, antenatal care, and place of delivery were associated with the uptake of HepB-BD. Infants of mothers who had primary or no formal education were 4% less likely to be vaccinated compared with infants of mothers with secondary or higher education (aRR=0.96, 95%CI=0.92-0.99). Infants of HepB-negative mothers who received antenatal care (aRR=1.23, 95%CI=1.16-1.31) or had an institutional delivery were more likely (aRR=1.29, 95%CI=1.23-1.36) to receive HepB-BD compared with infants whose mothers that did not receive antenatal care or have an institutional delivery, respectively.

## Discussion

This study assessed the coverage of HepB-BD and its associated factors among infants of HepB-positive and HepB-negative mothers in Nigeria. The HepB-BD coverage was 64% and there was no significant difference in the likelihood of infants of HepB-positive or HepB-negative mothers receiving HepB-BD. In HepB-positive mothers, ≥4 previous pregnancies or receiving antenatal care was associated with a greater likelihood of HepB-BD uptake, while < 20 years old was associated with a lower likelihood. However, in HepB-negative mothers, secondary or higher education attainment, antenatal care, or institutional delivery was associated with a greater likelihood of HepB-BD uptake. These findings highlight the need to improve HepB-BD coverage, particularly among infants exposed to HepB who are at increased risk of perinatal transmission of HepB.

The HepB-BD coverage in this study compares with the coverage of 68% reported among infants 12-24 months for Benue State in the 2018 Nigeria Demographic and Health Survey [12]. Although the HepB-BD coverage in Benue State is higher than the national average (52%), there is still a need to ensure more infants are vaccinated. According to the 2018 NDHS, the coverage of HepB-BD in Benue State was much lower than bacillus Calmette Guerin (BCG) (82%), which is also an injectable vaccine given at birth [12]. While the reasons for the wide disparity are not known, the limited availability of HepB vaccines in health facilities may be a contributing factor [14]. Providers may also not be aware of the significance of HepB vaccines or may lack the required skills to deliver the service [24,25].

Despite the recommendation for routine HepB screening of all pregnant women in Nigeria, many women go through antenatal care without being screened [26]. Community-based screening to reach pregnant women who do not attend health facilities for antenatal care has also been limited [27]. Although the national guidelines for hepatitis control in Nigeria specify universal HepB-BD, preferably within 24 hours of birth and not exceeding 2 weeks after birth, knowing the status of the mothers can further help in HepB prevention among infants at risk, as additional interventions such as maternal antiviral prophylaxis may be required, depending on the virological and serological status of the mothers [28]. As part of the HBI program, pregnant women were screened in the communities and those who had a positive test were referred to health facilities for standard care. Contrary to the authors´ expectations, there was no significant difference in HepB-BD coverage between those with positive and negative HepB tests. It is possible that some of the HepB-positive women did not present at the health facilities following the referral as a result of low-risk perception, denial, or stigma and discrimination [29-31]. This finding may also suggest that HepB-exposed infants are not prioritized and actively followed up by healthcare providers to ensure they receive HepB-BD.

Consistent with similar childhood vaccine studies in Nigeria [13,15,20-22], education, antenatal care, and institutional delivery were associated with HepB-BD in this study. Educated women are more likely to be aware of the HepB vaccine and its benefits, and perhaps be less hesitant [13]. Similarly, women who attend antenatal care are more likely to receive information about HepB vaccination, which may positively influence the uptake of vaccination. Institutional delivery may be associated with the uptake of HepB-BD because vaccines are more likely to be available at a health facility compared to homes or other places of delivery.

However, the results showed that the factors associated with HepB-BD varied by HepB status with only antenatal care common to the two groups. The reasons for the differences in the associated factors by HepB status are not clear but may be due to psychological and social problems that may follow the diagnosis of HepB [29]. Younger age was associated with the uptake of HepB-BD among positive mothers, whereas there was insufficient evidence to suggest any association between age and HepB-BD among Hep B-negative mothers. Compared to older women, younger HepB-positive mothers may have received more attention, and perhaps they are more easily influenced by healthcare providers and family members to accept vaccination. Similarly, among HepB-positive mothers, those with no previous pregnancies had significantly lower uptake, while it was not significantly associated with HepB-BD among negative mothers. Women who have never been pregnant and delivered are not likely to have experienced HepB-BD, hence they may be hesitant.

The strengths of this study include the large number of participants and the longitudinal design of the HBI program, where HepB status was determined before childbirth. The HBI was also conducted in about half of the 23 LGAs in Benue State for representativeness. However, the study has a few limitations. The coverage of HepB-BD was self-reported, and some participants may have given inaccurate reports. We also did not assess the timeliness of HepB-BD, which is critical to its effectiveness. Future studies may consider exploring the timeliness of HepB-BD among HepB-exposed and -unexposed infants. More qualitative studies are also needed to better understand the barriers and facilitators to HepB-BD vaccination from both providers´ and clients´ perspectives.

## Conclusion

The coverage of HepB-BD is suboptimal and similar among infants of HepB-positive and HepB-negative mothers in our study. However, the study findings suggest that the factors associated with HepB-BD vary by HepB status. While there is a need to improve universal coverage of HepB-BD, HepB-exposed infants must be identified through maternal screening and given HepB-BD to prevent perinatal transmission. This will require improving antenatal HepB screening, capacity building for healthcare workers, and ensuring accessibility and availability of HepB-BD [32]. Effective strategies to reach infants delivered at home are also urgently needed. This may include establishing a strong referral network between the communities and health facilities or community delivery of HepB-BD.

### What is known about this topic

- *Nigeria has the largest burden of hepatitis B (HepB) in Africa;*
- *Nigeria offers universal hepatitis B birth-dose vaccine (HepB-BD) for the prevention and control of perinatal transmission of HepB, however the coverage remains low*.

### What this study adds

- *The study shows that the sub-optimal coverage of HepB-BD is similar among HepB-positive and HepB-negative mothers in Benue State, Nigeria;*
- *The study highlights the need to improve HepB-BD uptake, particularly among HepB -exposed infants who are at risk of perinatal transmission of HepB*.

## References

[1] Nelson NP, Easterbrook PJ, McMahon BJ (2016). Epidemiology of hepatitis b virus infection and impact of vaccination on disease. Clin Liver Dis.

[2] World Health Organization (2019). Hepatitis B.

[3] Ott JJ, Stevens GA, Groeger J, Wiersma ST (2012). Global epidemiology of hepatitis B virus infection: New estimates of age-specific HBsAg seroprevalence and endemicity. Vaccine.

[4] Razavi-Shearer D, Gamkrelidze I, Nguyen MH, Chen DS, Van Damme P, Abbas Z (2018). Global prevalence, treatment, and prevention of hepatitis B virus infection in 2016: a modelling study. Lancet Gastroenterol Hepatol.

[5] Franco E, Bagnato B, Marino MG, Meleleo C, Serino L, Zaratti L (2012). Hepatitis B: Epidemiology and prevention in developing countries. World J Hepatol.

[6] Ranger-Rogez S, Denis F (2004). Hepatitis B mother-to-child transmission. Expert Rev Anti Infect Ther.

[7] Shiraki K (2000). Perinatal transmission of hepatitis B virus and its prevention. J Gastroenterol Hepatol.

[8] World Health Organization (2009). Hepatitis B vaccines. Wkly Epidemiol Rec.

[9] National AIDS/STIs Control Programme, Federal Ministry of Health (2016). National guidelines for the prevention, care and treatment of viral hepatitis B & C in Nigeria, Abuja.

[10] Keane E, Funk AL, Shimakawa Y (2016). Systematic review with meta-analysis: the risk of mother-to-child transmission of hepatitis B virus infection in sub-Saharan Africa. Aliment Pharmacol Ther.

[11] National AIDS/STIs Control Programme, Federal Ministry of Health (2016). National strategic plan for the control of viral hepatitis in Nigeria (2016-2020). Abuja.

[12] Nigeria Population Commission (2019). Nigeria demographic and health survey 2018. NPC, ICF.

[13] Okenwa UJ, Dairo MD, Bamgboye E, Ajumobi O (2020). Maternal knowledge and infant uptake of valid hepatitis B vaccine birth dose at routine immunization clinics in Enugu State-Nigeria. Vaccine.

[14] Okenwa UJ, Dairo MD, Uba B, Ajumobi O (2019). Maternal reasons for non-receipt of valid Hepatitis B birth dose among mother-infant pairs attending routine immunization clinics, South-east, Nigeria. Vaccine.

[15] Olakunde BO, Adeyinka DA, Olakunde OA, Ogundipe T, Oladunni F, Ezeanolue EE (2022). The coverage of hepatitis B birth dose vaccination in Nigeria: Does the place of delivery matter?. Trans R Soc Trop Med Hyg.

[16] Ezeanolue EE, Obiefune MC, Ezeanolue CO, Ehiri JE, Osuji A, Ogidi AG (2015). Effect of a congregation-based intervention on uptake of HIV testing and linkage to care in pregnant women in Nigeria (Baby Shower): A cluster randomised trial. Lancet Glob Health.

[17] Ezeanolue EE, Obiefune MC, Yang W, Obaro SK, Ezeanolue CO, Ogedegbe GG (2013). Comparative effectiveness of congregation-versus clinic-based approach to prevention of mother-to-child HIV transmission: study protocol for a cluster randomized controlled trial. Implement Sci.

[18] National Agency for Food and Drug Administration and Control (2018). Registered products database.

[19] Eze P, Agu UJ, Aniebo CL, Agu SA, Lawani LO, Acharya Y (2021). Factors associated with incomplete immunisation in children aged 12-23 months at subnational level, Nigeria: a cross-sectional study. BMJ Open.

[20] Adegboye OA, Kotze D, Adegboye OA (2014). Multi-year trend analysis of childhood immunization uptake and coverage in Nigeria. J Biosoc Sci.

[21] Olorunsaiye CZ, Degge H (2016). Variations in the Uptake of Routine Immunization in Nigeria: Examining Determinants of Inequitable Access. Glob Health Commun.

[22] Chidiebere ODI, Uchenna E, Kenechi OS (2014). Maternal sociodemographic factors that influence full child immunisation uptake in Nigeria. South African J Child Health.

[23] Antai D (2012). Gender inequities, relationship power, and childhood immunization uptake in Nigeria: A population-based cross-sectional study. Int J Infect Dis.

[24] Djaogol T, Coste M, Marcellin F, Jaquet A, Chabrol F, Giles-Vernick T (2019). Prevention and care of hepatitis B in the rural region of Fatick in Senegal: a healthcare workers´ perspective using a mixed methods approach. BMC Health Serv Res.

[25] Adeyemi AS, Afolabi AF, Adeomi AA (2014). Hepatitis B Virus (HBV) Infection in Pregnancy: Knowledge and Practice of Care Providers in Nigeria. Open J Obstet Gynecol.

[26] Olakunde BO, Adeyinka DA, Ndukwe CD, Oladele TT, Yahaya HB, Ijaodola OA (2021). Antenatal hepatitis B screening in Nigeria: A comparative analysis with syphilis and HIV. Int J STD AIDS.

[27] Olakunde BO, Adeyinka DA, Olakunde OA, Uthman OA, Bada FO, Nartey YA, Sonderup MW (2021). A systematic review and meta-analysis of the prevalence of hepatitis B virus infection among pregnant women in Nigeria. PLoS One.

[28] World Health Organization (2020). Prevention of mother-to-child transmission of hepatitis B virus: Guidelines on antiviral prophylaxis in pregnancy. Geneva.

[29] Adjei CA, Naab F, Donkor ES (2017). Beyond the diagnosis: a qualitative exploration of the experiences of persons with hepatitis B in the Accra Metropolis, Ghana. BMJ Open.

[30] Adjei CA, Stutterheim SE, Naab F, Ruiter RAC (2020). “To die is better than to tell”: reasons for and against disclosure of chronic hepatitis B status in Ghana. BMC Public Health.

[31] Mutyoba JN, Surkan PJ, Makumbi F, Aizire J, Kirk GD, Ocama P (2021). Hepatitis B birth dose vaccination for newborns in Uganda: A qualitative inquiry on pregnant women´s perceptions, barriers and preferences. J Virus Erad.

[32] Boisson A, Goel V, Yotebieng M, Parr JB, Fried B, Thompson P (2022). Implementation Approaches for Introducing and Overcoming Barriers to Hepatitis B Birth-Dose Vaccine in sub-Saharan Africa. Glob Health Sci Pract.

